# Identifying candidate genes affecting developmental time in *Drosophila melanogaster*: pervasive pleiotropy and gene-by-environment interaction

**DOI:** 10.1186/1471-213X-8-78

**Published:** 2008-08-08

**Authors:** Julián Mensch, Nicolás Lavagnino, Valeria Paula Carreira, Ana Massaldi, Esteban Hasson, Juan José Fanara

**Affiliations:** 1 Departamento de Ecología, Genética y Evolución, Facultad de Ciencias Exactas y Naturales, Universidad de Buenos Aires, Argentina

## Abstract

**Background:**

Understanding the genetic architecture of ecologically relevant adaptive traits requires the contribution of developmental and evolutionary biology. The time to reach the age of reproduction is a complex life history trait commonly known as developmental time. In particular, in holometabolous insects that occupy ephemeral habitats, like fruit flies, the impact of developmental time on fitness is further exaggerated. The present work is one of the first systematic studies of the genetic basis of developmental time, in which we also evaluate the impact of environmental variation on the expression of the trait.

**Results:**

We analyzed 179 co-isogenic single *P[GT1]-*element insertion lines of *Drosophila melanogaster *to identify novel genes affecting developmental time in flies reared at 25°C. Sixty percent of the lines showed a heterochronic phenotype, suggesting that a large number of genes affect this trait. Mutant lines for the genes *Merlin *and *Karl *showed the most extreme phenotypes exhibiting a developmental time reduction and increase, respectively, of over 2 days and 4 days relative to the control (a co-isogenic *P*-element insertion free line). In addition, a subset of 42 lines selected at random from the initial set of 179 lines was screened at 17°C. Interestingly, the gene-by-environment interaction accounted for 52% of total phenotypic variance. Plastic reaction norms were found for a large number of developmental time candidate genes.

**Conclusion:**

We identified components of several integrated time-dependent pathways affecting egg-to-adult developmental time in *Drosophila*. At the same time, we also show that many heterochronic phenotypes may arise from changes in genes involved in several developmental mechanisms that do not explicitly control the timing of specific events. We also demonstrate that many developmental time genes have pleiotropic effects on several adult traits and that the action of most of them is sensitive to temperature during development. Taken together, our results stress the need to take into account the effect of environmental variation and the dynamics of gene interactions on the genetic architecture of this complex life-history trait.

## Background

Development is a variable and time-dependent process that can be thought of as mapping genotypes on to adult phenotypes [1,2]. The time elapsed from the embryo to the reproductive phase, commonly known as developmental time (DT), is a trait of great relevance to fitness in all organisms [3]. In particular, *Drosophila *species occupy ephemeral habitats, such as rotting fruits that may result in selection for rapid development. Quoting Gould's *Ontogeny and Phylogeny: *"The timing of maturation is a primary variable in setting life history strategies. We have a prima facie case for ascribing direct significance to the change in developmental timing itself, not only to its morphological consequences"[4]. In addition, changes in the timing of developmental processes – heterochrony- may partly account for many evolutionary changes we observe because of genetic correlations of DT with other life-history related traits. Incidentally, heterochrony can also proceed by truncation in addition to retardation/acceleration of developmental processes [5]. The genes that control development are broadly shared among distantly related groups, and some of the pathways are surprisingly similar in superficially different organisms [6,7]. In this sense, experimental studies of egg-to-adult heterochrony at the molecular level can provide insights into the evolutionary processes by uncovering the genetic basis of ecologically relevant adaptive traits.

DT is a complex trait that displays considerable genetic variation in natural populations [8,9]. Laboratory selection for reduced DT triggered a negatively correlated response of other life-history traits, such as adult weight at eclosion [10], adult size [10-13], pre-adult survival [12,13] and longevity [14]. The reduction of adult fitness as a consequence of the acceleration of DT is a pattern called *fast development syndrome *and it illuminates the direct connection of the pre-adult and adult stages through energetic trade-offs [14]. These studies highlight the negative genetic correlations between DT and a large number of adult life-history traits, suggesting that genes involved in early development may also have pleiotropic effects on adult traits. However, specific genes contributing to variation in DT, and its correlations with other life-history traits, remain largely unknown.

The analysis of the genetic architecture of a trait requires not only the identification of the set of genes involved in its expression, but also their response to environmental variation (phenotypic plasticity) [15-17]. Phenotypic plasticity refers to the ability of a genotype to produce alternative morphological, behavioral and physiological characteristics in response to environmental conditions [18-21]. Temperature is an important environmental factor that affects the developing organism and surely has played a major role during the evolution of developmental traits in ectotherms [22-24]. Indeed, ectotherms must adapt their developmental program to a wide range of environmental temperatures during the day and also in different seasons along the year.

*Drosophila *brings an impressive toolkit for dissecting multiple interacting loci with individually small and environmentally sensitive effects that affect complex traits [15]. *P*-element mutagenesis is an effective strategy for studying the effects of single mutations on complex phenotypes [15]. A comprehensive understanding of DT requires the knowledge of: (1) the identities of the genes involved in the expression of the phenotype, (2) the underlying biological processes and molecular pathways, (3) the pleiotropic effects on adult traits and (4) the genetic basis of phenotypic plasticity in response to environmental variation.

Here we report a study of DT that reveals a large number of heterochronic mutants, most of which retard the time to reach the adult stage. The fastest developing line in our survey is a mutant of *Merlin*, a gene encoding a protein that regulates cell proliferation in developing imaginal discs. Interestingly, *Merlin *is the homolog of the human tumor suppressor gene *Neurofibromatosis Type 2 *(NT2) involved in deregulation of cell proliferation in pathologies of the central nervous system [25]. In addition, we provide evidence for extensive pleiotropic effects of growth control genes on adult traits. Finally, we discovered several candidate genes involved in the plastic response to temperature variation.

## Results

### Pattern of variation among co-isogenic *P[GT1] *insertion lines

**Figure 1:**
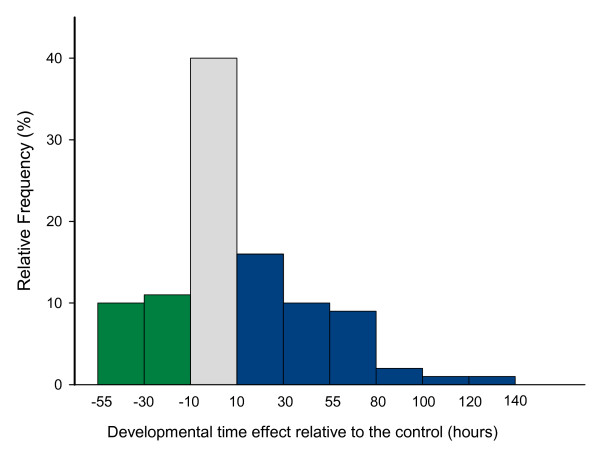
**Frequency distribution of mutational effects on developmental time of 179 *P[GT1] *insertion lines. **Grey bar represents lines showing similar effects relative to the control line (40%); green and blue bars represent lines showing a reduction (20%) and an increase (40%) in DT, respectively. Values in x-axis show the limits of each DT intervals.

Substantial phenotypic variation among lines was observed for DT (Figure 1), wherein the most commons effect was an increase of DT relative to the control. This is not unexpected as the most likely effect of mutations on a fitness-related trait is reasonable to be deleterious, although, we found a significant positive correlation (r: 0.3, p < 3 × 10^5^) between DT and viability [see Additional file 1]. This pattern revealed that the result observed of the heterochornic effect of the *P[GT1] *insertion lines can not be explain as a consequence of unfit flies. The most extreme phenotypes were exhibited by lines BG01543 and BG01412. The former developed 60 and 50 hours (males and females, respectively) faster and the latter 119 and 146 hours (males and females, respectively) slower than the control. We observed a significant line effect indicating mutational variance among single *P*-element insertion lines (Table 1). Indeed, the line effect accounts for 84% of total variance, five times higher than the error. In the ANOVA, differences between sexes were also significant, with males developing faster than females. In addition, the contribution of the line-by-sex interaction to the total phenotypic variance was not significant.

**Table 1:** Analysis of variance of mutational effects on developmental time.

Source	d.f.	*F*	*P*	σ^2^(%)
Line	178	54.68	<0.0001	84
Sex	1	46.87	<0.0001	Fixed
Line × Sex	178	0.76	0.99	0
Error	1066			16

d.f.: degree of freedom; σ^2^: component variance.

### Identification of co-isogenic *P[GT1] *insertion lines affecting DT and functional analysis

*Dunnett *analyses revealed that 107 out of 179 lines (60%) differed significantly from the control. Mean values of all significant *P[GT1] *insertion lines tested at 25°C are shown in Additional file 2. In seventy-four lines (40%) the insertion of the *P*-element caused an increase in DT, while in the rest of the significant lines DT was shortened as compared to the control (20%) (Figure 1). Those lines that exhibited significant DT differences relative to the control (heterochronic mutants) were considered as lines bearing an insertion in a candidate DT gene. Among fast developing lines, BG01543 and BG01902 stand out, since DT was reduced in each by more than 2 days. The *P*-element in line BG01902 disrupts *mastermind *(*mam*), a gene encoding a glutamine-rich nuclear protein [26] that is a positive transcriptional regulator of the *Notch *signaling pathway [27-30]. *Notch *is a central element in the cell signaling mechanism that controls a broad spectrum of cell fate choices during development across metazoans [31-33]. Interestingly, in humans, abnormalities in *Notch *signaling have been linked to a number of diseases, such as T cell acute lymphoblastic leukemia [34] and aortic valve disease [35]. In line BG01543, the *P*-element insertion occurs in *Merlin *(*Mer*), a well-known negative regulator required for cell proliferation in developing imaginal discs of *Drosophila *[25]. It is a component of the *Hippo *signaling pathway, which is essential for the regulation of organ size during development [36]. *Mer *might control tissue growth by regulating endocytosis of membrane receptors in imaginal epithelia [37]. Interestingly, *Mer *is the homolog of the human tumor suppressor gene *Neurofibromatosis Type 2 *(NT2) involved in the de-regulation of cell proliferation in tumors of central nervous system pathologies [25,38].

Another gene involved in a cell proliferation pathway identified in our screen is *forkhead box, sub-group O (foxo)*. The insertion line BG01573 bearing a *P*-element in *foxo *increased DT by 24 hours in both sexes. *foxo *is a key regulatory component of the insulin-signaling pathway, which in *Drosophila *regulates the control of growth size of cells, organs, and the entire body in response to nutrient availability [39]. Puig *et al*. [40] established that *foxo *activates transcription in downstream as well as upstream targets of this signaling cascade by a transcriptional feedback mechanism that regulates cell growth and proliferation. It has been suggested that the insulin-signaling pathway regulates cell proliferation in imaginal discs, though the duration of the proliferation phases are controlled by Juvenile Hormone (JH) and ecdysteroids, that are themselves unaffected by the insulin-signaling pathway [41]. Premature ecdysone release leads to the rapid development of small adults, while the delay in ecdysone release extends developmental time and produces larger adults [42]. Recent studies have shown that the activity of the insulin-signaling pathway in the prothoracic gland modulates ecdysone release and influence both the length and the rate of larval growth [43-45]. It has been suggested that the neuropeptide *Amnesiac (Amn) *participates in regulating ecdysone synthesis in the prothoracic gland of Drosophila [43]. Interestingly, the *P*-element insertion mutant at *Amn *(line BG02286) also extended DT by more than two days in both sexes [see Additional file 2]. However, ecdysone can directly inhibit insulin and growth in the fat body and other peripheral tissues, an event that triggers metamorphosis. Furthermore, the ecdysone suppression of growth rate is lost in *foxo *mutants [45], indicating complex cross-talk between the ecdysone and the insulin signaling pathway during *Drosophila *development.

Our survey revealed extreme heterochronic mutants, particularly in the direction of increased DT [see Additional file 2]. For instance, the *P*-element insertion in the gene *Karl *(BG01010) extended DT by more than 100 hours in both sexes. The molecular function inferred from protein sequence suggests that *Karl *is a retinol-binding protein with no clear association to a biological process. In line BG00372 we registered a delay of 93 hours and 108 hours for males and females, respectively. In this case the *P*-element has inserted 1498 bp downstream of the 3' end of the *CG1678 *gene. The molecular function and the biological processes in which this gene is involved are unknown. However, there is evidence that *CG1678 *interacts with other genes such as *And, ewg, CG1472, rl, Bsg25D, CG11275 *and *msb1l *[46]. Line BG01412 exhibited the most extreme phenotype in our screen, the *P*-element insertion was associated with an extended developmental time of 119 and 146 hours in males and females, respectively. Unfortunately, information about the nucleotide sequences flanking the *P*-element insertion site is not available.

Candidate DT genes are involved in a wide range of biological processes according to their gene-ontology (GO) terms (Table 2). Interestingly, DT genes not only are involved in processes associated to organismal development but also to biosynthetic and cellular metabolic processes. We did not find significant differences between groups of candidate genes accelerating development vs those that extended development in the distribution among GO terms, suggesting that similar ontogenetic pathways may be responsible for both types of heterochronic phenotypes.

**Table 2:** Distribution of candidate DT genes among biological process GO terms.

Biological process GO terms	% of genes
Cellular metabolic process	57.9
Multicellular organismal development	39.5
Macromolecule metabolic process	36.8
Anatomical structure development	34.2
Regulation of biological process	31.6
Cellular developmental process	31.6
Cell communication	29.0
Biosynthetic process	23.7
Cellular component organization and biogenesis	18.4
Sexual reproduction	18.4
Establishment of localization	18.4
Nitrogen compound metabolic process	10.5
Behavior	10.5
Response to stress	10.5
Cell proliferation	10.5
Cell adhesion	10.5
Cell cycle	7.9

Genes are distributed in a non-exclusive manner (i.e. a given gene might be associated to more than one GO term, see text for more details). The percentage of genes related to each GO term is shown.

### Candidate genes for plasticity

**Figure 2:**
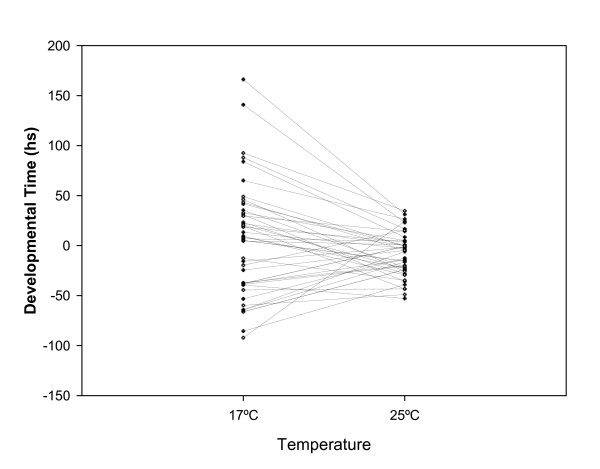
**Reaction norms of 45 *P[GT1] *insertion lines tested at 17°C and 25°C.** Values are shown as the deviation of the insert line mean from contemporaneous control line.

Growth and development of ectotherms are determined in part by their thermal environment [22,23]. In particular, temperature during ontogeny exerts a strong influence shaping the evolution of larval traits [24,47,48]. In fact, phenotypic responses result of adaptation to different thermal environments and/or may be an unavoidable consequence of the effect of temperature on the organism's physiology during development [49]. In this context, the pattern of phenotypic effects of *P*-element insertion lines reared at different developmental temperatures would provide new insights in the study of phenotypic evolution of larval traits. The ANOVA showed that differences among lines and between thermal treatments were significant (Table 3). More importantly, our screen revealed that the line-by-sex and the line-by-temperature interactions were also highly significant, indicating that the behavior of each line depended on the temperature at which it was reared and the sex. However, there was a large difference in the magnitude of these genotype-by-environment interactions. The former accounts for only 1% of the total phenotypic variance (a percentage similar to that obtained in the 25°C assay) while the line-by-temperature interaction explained 52% of the variation. Moreover, note that in the general assay, the percentage of total phenotypic variance explained by differences among lines was 84%, whereas in our assays of phenotypic plasticity this percentage dropped to 30%. It may be argued that part of the effect was obscured by the high value of the line-by-temperature interaction term. This observation opens an excellent opportunity for studying the genetic basis of phenotypic plasticity of developmental time. Thus, we decided to analyze whether the significant line-by-temperature interaction can be explained by changes in magnitude of among-line variance across thermal treatments or changes in the rank order among lines, i.e. a cross-temperature genetic correlation lower than unity. Our results showed that about half of the interaction variance can be explained by a greater among-line variance observed at 17°C than 25°C and the other half by temperature-specific effects on DT of the lines (Figure 2).

**Table 3:** Analysis of variance of mutational effects on developmental time among lines tested at different thermal treatments.

Source		d.f.	*F*	*P*	σ^2^(%)
Line		41	2.11	0.0085	30
Sex		1	8.82	0.0047	Fixed
Thermal Treatment		1	4.56	0.03	Fixed
Line × Sex		41	1.73	0.041	1
Line × Thermal Treatment		41	41.4	<0.0001	52
Sex × Termal Treatment		1	9.26	0.0038	Fixed
Line × Sex × Thermal Treatment		41	0.56	0.9886	0
Error		479			17

d.f.: degree of freedom;: σ^2 ^component variance.

**Figure 3:**
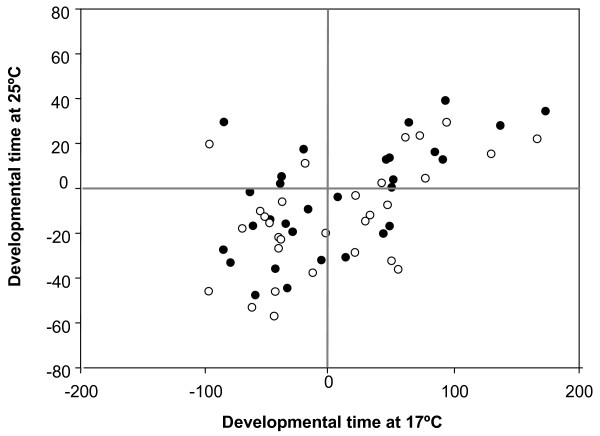
**Correlation of 30 *P[GT1] *heterochronic mutants tested at 17°C and 25°C. **Values are shown as the deviation of the insert line mean from contemporaneous control line for each sex separately; males (solid circles) and females (open circles).

**Figure 4:**
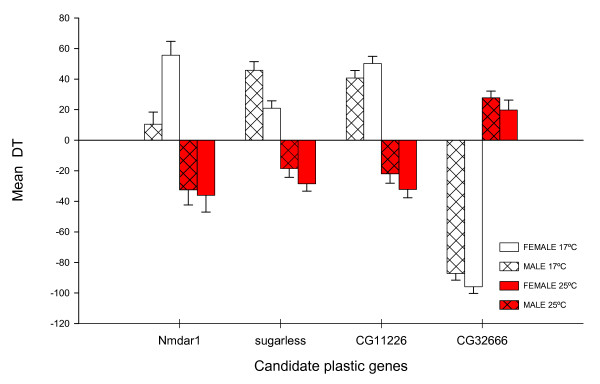
**Heterochronic mutants having opposite phenotypic effects between thermal treatments. **17°C assay is represent with open bars and 25°C assay with red bars. Males are represented with striped bars and females with open bars.

Thirty out of 42 lines tested at 17°C and 25°C showed significant differences relative to the control at one or both temperatures (Table 4). In addition, DT measured at different temperatures were positive correlated for the set of 30 heterochronic lines in both sexes (males: r: 0.61, p < 0.05; females: r: 0.55, p < 0.05; Figure 3). These results imply that most of the mutations that affected DT at both temperatures did so in the same phenotypic direction (either increasing or decreasing DT). Forty seven percent of the lines showed this pattern (bottom left and top right quadrants in Figure 3). Nevertheless, we also identified a set of lines in which the effect of the mutation on DT was temperature-dependent, suggesting that the mutated loci may be possible candidates for temperature plasticity genes. Indeed, forty percent of the lines affected DT in only one of the temperatures. We observed three lines showing a heterochronic phenotype only at 25°C. The line bearing an insertion in *CG14478 *is an example of this pattern. In contrast, nine lines showed a heterochronic phenotype only at 17°C. For example, the insertion in *Imp *exhibited significant differences at 17°C in both sexes, but not at 25°C. Surprisingly, four lines (13%) affected DT in opposite phenotypic directions in the two rearing temperatures. On the one hand, line BG02159, bearing a mutation in *CG32666*, decreased and increased DT at 17°C and 25°C, respectively. On the other hand, mutant lines for genes *Nmdar*, *sugarless *and *CG11226 *exhibited the opposite pattern (Figure 4).

**Table 4:** Effects of significant *P[GT1] *insertion lines screened at different thermal treatments.

Line	Gene	Males 17°C	Females 17°C	Males 25°C	Females 25°C	S	T	S × T
BG00369	*CG13334*	-34.25*	-44.73**	-44.50***	-56.99**	N	***	N
BG00373	*CG11226*	84.18***	72.11***	16.22*	23.52***	N	***	N
BG00386	*Nmdar1*	NS	54.81**	-30.73***	-36.13***	N	***	*
BG00737	*Hsp27*	-64.24***	-55.94***	NS	NS	**	***	N
BG00930	no sequence	45.08***	NS	NS	NS	**	***	N
BG01028	*CG33260*	-35.5*	-41.24**	NS	-21.81**	N	**	N
BG01214	*CG10064*	47.86***	NS	-16.82*	-28.59***	***	***	N
BG01339	*clt*	92.63***	93.64***	39.14***	29.46***	N	***	N
BG01354	*CG30492*	NS	41.53**	NS	NS	**	***	N
BG01488	*msi*	NS	-41.18**	-19.40**	-26.76***	N	*	N
BG01672	*CG14591*	-85.53***	-48.45**	-27.40**	NS	N	***	N
BG01716	*paps*	-38.8*	-39.38*	NS	-22.74**	*	***	N
BG01726	*CG11382*	-39.82**	-38.27*	NS	NS	N	***	N
BG01735	*bib*	NS	NS	NS	-19.91**	N	N	*
BG01763	*CG33960*	173***	166.28***	34.41***	NS	N	**	N
BG01780	*CG11226*	42.8**	49.41***	-20.18*	-32.31***	N	***	N
BG01822	*Imp*	-47.95**	-52.38**	NS	NS	N	***	N
BG01902	*mam*	-59.92***	-62.62***	-47.64***	-53.05***	N	*	N
BG01912	*pxb*	-79.85***	-97.30***	-33.10***	-45.92***	N	***	N
BG01990	*CG30492*	-43.82**	-43.86**	-35.87***	-46.05***	N	N	N
BG02042	*eas*	47.78**	NS	NS	NS	N	***	N
BG02088	*CG15309*	49.55**	46.28**	NS	NS	N	***	N
BG02102	*CG13434*	-61.79***	-70.34***	-16.73*	-17.89*	*	***	N
BG02157	*CG8177*	90.67***	76.60***	NS	NS	N	***	N
BG02159	*CG32666*	-85.12***	-96.73***	29.51***	NS	N	***	*
BG02239	*CG11550*	63.36***	60.57***	29.40***	22.72***	N	***	N
BG02462	*CG6301*	50.77***	38.37*	NS	NS	*	***	N
BG02690	*CG14478*	NS	NS	-32.02***	-37.68***	N	***	N
BG02747	*rut*	136.73***	129.40***	28.00***	NS	N	***	N
BG02823	*scyl*	NS	NS	17.44*	NS	N	***	N

Values are shown as the deviation of the insert line mean from contemporaneous control line. Asterisks in columns S, T and S × T represent the quantitative effect of Sex, Thermal treatment and Sex-by-Thermal treatment in response to environmental variation (see materials and methods for detail statistical treatment). *p < 0.05; **p < 0.01; ***p < 0.001; NS: no significant.

## Discussion

### Genetic architecture of developmental time

The genetic architecture of a trait determines the variational properties of the phenotype, that is, its evolutionary potential. Our study revealed 116 heterochronic independent mutants involved in the expression of DT (107 lines showed a mutant phenotype at 25°C, while 9 lines were heterochronic only at 17°C). In this sense, we were capable to identify components of different kind of pathways involved in DT expression. On the one hand, some components correspond to molecular mechanisms directly involved in timekeeping processes, such as, the ecdysone and the insulin-signaling pathway [41,50,51]. It has been established that environmental inputs during ontogeny influences multiple steps of the these kind of heterochronic gene pathways, such as, the nutrition status and the light: dark regimes. A paradigmatic example of this kind of environmental input is the circadian rhythm *period *mutants that alter DT in *Drosophila *[52]. On the other hand, we also observed heterochronic phenotypes that seem to be a by-product of the disruption of a pathway that is not explicitly involved in the control of temporal process but plays a major role in organ growth This is the case of *Merlin*, a negative regulator of the *Hippo *signaling pathway, required for cell proliferation in developing imaginal discs. Interestingly, *Merlin *is a component of a tumour-suppressor network associated with human tumorigenesis [36]. All in all, our results support the hypothesis raised by Moss [53] that timing of development is control directly and indirectly by different ontogenetic pathways.

Since the same co-isogenic lines were also screened for abdominal and sternopleural bristle number, starvation resistance and olfactory behavior [17,54,55] we also examined whether DT candidate genes have pleiotropic effects on adult fitness-related traits. Several DT heterochronic lines (genes) affect starvation resistance (4 genes), bristle number (8 genes) and olfactory behavior (13 genes) [see Additional file 3]. Once again, *Merlin *seems to plays a role not only in egg-to-adult DT but also in starvation resistance and adult olfactory behavior. In BG01543 (*Mer *mutant), the *P[GT1] *insertion in exon 1 caused a reduction in the RNA expression levels in embryos, third instar larvae and adults but not in the pupal stage compared to the control [17] indicating that anomalies caused by the disruption of *Merlin *on DT took place before metamorphosis. Since insects do not grow as adults, their final size is a product of growth rate and length at larval stages [56]. Surprisingly, the mutated *Merlin *line did not differ significantly from the control in several morphological traits such as face width, head width, thorax length and wing size suggesting a decoupled mechanism of timing control and size development (Carreira, Mensch & Fanara, unpublished results). Regarding the pleiotropic effect of *Mer *on starvation resistance and adult olfactory behavior, it is not easy to discern whether aberrant phenotypes are the consequence of early developmental events or are directly related to the functional disruption of the gene product in the adult. As another example, the insertion line for *tramtrack *(*ttk*), a transcription repressor gene involved in cell-fate determination [57], increased DT by two days in both sexes and also affected starvation resistance and olfactory behavior. These interesting examples constitute clear evidence of an intricate network of pleiotropic effects of key developmental genes throughout the life-cycle of a holometabolous organism. Such pervasive pleiotropy of key genes that control development, affecting early and late fitness-related traits, show an integrated picture of the evolution of life-history traits at the molecular level.

Regarding the effect of sex, males developed significantly faster than females, an observation that is quite striking since it is at odds with extensive literature showing that *Drosophila *females usually reach adulthood before males in the species studied so far [58]. Moreover, a recent study by Paranjpe *et al*. [59] showed that females developed faster than males in *D. melanogaster*. However, some differences between our study and Paranjpe *et al.'*s in terms of the experimental design are worth mentioning. Paranjpe *et al*. found that females developed faster than males under several photoperiod regimes during ontogeny, including 12:12 L:D (the regime performed in our study). Nevertheless, we must note that different genetic backgrounds were analyzed in these two studies. Indeed, our control line, which shares the same genetic background with all mutant lines tested, did not show any sign of sexual dimorphism in DT, stressing the need to take into account the effect of the genetic background on the expression of the analyzed trait since genetic background × gene interactions are known to be quite pervasive [60,61]. At the same time the null input of the line-by-sex interaction to the total phenotypic variance adds a complementary aspect to the discussion of the absence of sexual dimorphism for DT. In the last few years a similar set of coisogenic insertion lines was studied for several adult traits for which the line-by-sex interaction explained significant portions of variance. In this context, our DT survey yielded the lowest value in this category, followed by sternopleural bristle number (5%), adult odor-behavior (13%), abdominal bristle number (21%) and starvation resistance (66%) [17,54,55]. Among the heterochronic mutants, nine had a male-specific effect and in seven only female DT was affected. It is important to note that none of the lines showed opposite phenotypic effects across sexes, meaning that we did not find any lines that decreased DT in one sex and increased the trait in the other. In conclusion, our results indicate that there are features of the genetic architecture of DT that are highly conserved in both sexes.

### Gene-by-environment interaction

Although the multiple interacting genes affecting complex traits can readily be dissected, how much genotype-environment interactions contribute to variation in these traits remains elusive. The quantitative dissection of developmental time reported here reveals that almost all heterochronic lines tested at different developmental temperature presented an effect with quantitative variation between temperatures, a remarkable consequence of the large gene-by-environment interaction (Table 4). The most striking examples of these developmental reaction norms are the mutant lines for genes *CG11226, CG32666, Nmdar1*, and *sugarless *(Figure 4). CG32666 gene product is a receptor signaling protein with serine/threonine kinase activity, NMDAR1 is a ionotropic glutamate receptor and SUGARLESS is a protein with UDP-glucose 6-dehrygenase activity indicating that this routine cellular metabolic processes are not only involved in developmental time but also that are sensitive to environmental variation.

The part of the genetic diversity that has the potential to affect the phenotype, but that is not expressed under the current genotypic or environmental conditions, is referred to as "cryptic" or "hidden" genetic variation [62]. Molecular mechanisms responsible for this particular genetic variation include epistasis and genotype-by-environment interactions. In our case, in addition to the plastic developmental reaction norms, large input to the gene-by-environment interaction refers to the bigger variance magnitude at 17°C (Figure 2). It is possible that at 25°C genetic variation was buffered in comparison to the variation expressed at 17°C, an unusual rearing temperature for these lines. Taken together, our results highlight the large potential of the *Drosophila *genome to changes in this relevant environmental factor, although this genetic plasticity was exhibited by these particular inbred lines, a pattern that it is not necessarily true for outbred populations. Future efforts will focus on elucidating the molecular mechanisms controlling the temperature plastic response observed. Since in nature insects are exposed to a wide range of environmental temperatures during the day and also in different seasons along the year with local-specific thermal regimes, we are also interested in studying the evolutionary forces shaping the variation in the candidate DT genes.

## Conclusion

The study of heterochrony has played an important role in the intersection of evolution, genetics and developmental biology since the late nineteenth century. During the last decade the concept has been revitalized with studies at cellular and molecular level. Here we identified components of several integrated time-dependent pathways affecting egg-to-adult DT and also components of pathways that are not explicitly involved in the control of temporal processes. In this sense, we identified 116 egg-to-adult heterochronic mutants, most of them developed slower compared to the control. However, we identified a set of lines that developed faster by more than two days. This is the case of *Mer *mutant line, a gene involved in a cell proliferation pathway. *Mer *and others DT candidate genes have pleitropic effects on adult traits as well. Most of developmental time candidate genes were sensitive to the rearing temperature, a fact that stresses the need to take into account the effect of environmental variation on the genetic architecture of complex traits.

## Methods

### Drosophila stocks and Developmental time assays

We scored 179 homozygous viable *P[GT1] *insertion lines, contructed in a co-isogenic *Canton-S B *background [63] as part of the *Berkeley Drosophila Genome Project *(see Availability and requirements section for URL) for DT.

Batches of 30–40 P-element insertion lines were assessed simultaneously. To account for environmental variation in DT between batches, 8 replicated vials of the control strain (a co-isogenic P-element insertion free line with the same genetic background) was run in parallel with each batch. For each line, 300 pairs of sexually mature flies were placed in oviposition chambers for 8 hours. Eggs were allowed to hatch and batches of 30 first-instar larvae were transferred to culture vials containing a cornmeal-agar-molasses medium (4 replicates vial per line). Emerged flies from each vial were collected every 12 hours and sorted by sex. We estimated DT as the time elapsed since the transfer of first-instar larvae to the vials until adult emergence. Each vial was kept in an incubator at 25°C ± 0.5, under a 12:12 h light:dark photoperiod and at 60–70% of humidity. Thirteen lines with low viability (those that reached less than 50% of control pre-adult survival) were excluded from our survey to avoid the confusing effect of high pre-adult mortality with DT. A subset of 42 lines selected at random was also screened at 17 ± 0.5°C.

### Statistical analysis

#### Quantitative genetic analysis

Analysis of variance (ANOVA) was utilized to assess the magnitude of mutational variance for DT at each temperature. In order to include all lines tested in different batches, individual DT scores were expressed as deviations from the mean of their contemporaneous co-isogenic controls, separately for males and females. Three-way ANOVA was computed for each thermal treatment, following the mixed model: Y = μ + L + S + L × S + E, where μ is the overall mean, L is the random effect of line, S is the fixed effect of sex and E represents the error term. An ANOVA was used to assess the magnitude of mutational variance for DT induced by *P[GT1] *insertions. We also estimated the relative contribution of all random sources of variation (Line, Line × Sex, and error) to the total variance.

To identify which lines were responsible of the significant line or line-by-sex interaction, phenotypic differences between *P*-element insertion lines and the control were tested using *Dunnett *contrasts for each temperature and batch. Those lines that exhibited significant DT differences relative to the control (heterochronic mutants) were considered as lines bearing an insertion in a candidate DT gene.

#### Gene-by-thermal treatment analysis

In those lines screened at both temperatures (17 and 25°C) we studied the effect of phenotypic plasticity by thermal treatments following the mixed ANOVA model, Y = μ + L + S + T + L × S + L × T + S × T + L × S × T + E, where μ is the overall mean, L is the random effect of line, S is the fixed effect of sex, T is the fixed effect of thermal treatment and E represents the error term. Since significant G × E (genotype × environment) can arise due to: (1) differences among- lines variance in separate environments (change in scale) and/or (2) deviations from unity of the cross-environment genetic correlation (*rG *× *E *< 1, see below) (changes in rank order), the contribution of these two sources of variation was analyzed by means of the equation derived by Robertson [64], *V*_*G *× *E *_= [(σ_E1 _- σ_E2_)^2 ^+ 2σ_E1_σ_E2_(1 - *rG *× *E*)]/2, where *V*_*G *× *E *_is the G × E variance component, *rG *× *E *is the cross-environment genetic correlation and, σ_E1 _and σ_E2 _are the square roots of the among-line variance components of the two thermal environments studied [65]. The first term corresponds to differences in among-line variance while the second corresponds to deviations from the perfect correlation across environments (*rG *× *E *< 1). The cross-environment genetic correlation (*rG *× *E *< 1) is the genetic correlation of measurements in different environments and here reflects the degree in which the same genes control the expression across environments. *rG *× *E *was estimated as: *rG *× *E *= COV_E1E2_/σ_E1 _σ_E2_, where COV_E1E2 _is the covariance of lines in different environments and it was calculated as the covariance of the lines in different environments.

Finally, we studied the phenotypic change by the effect of the *P *element insertion in each line showing significant differences with respect to the control in at least one thermal assay. In this case we used the following fixed ANOVA model:

Y = μ + T + S + T × S + E, where T stands for the effect of thermal treatment, S for the effect of sex and E represents the error term. Those lines that exhibited significant differences in DT between thermal treatments (T factor) were considered as lines bearing a mutation in a candidate gene involved in the plastic response of DT to temperature variation.

All statistical analyses were performed using STATISTICA software packages (StatSoft, Inc. 1999, 2001).

#### Gene identification and functional analysis

In order to identify the mutated genes, nucleotide sequences flanking the *P*-element insertion in each candidate line were used to search for homologous regions in the *D. melanogaster *genome. Searches of homologous sequences were performed against Release 5 of the published *D. melanogaster *genomic sequence using the Pubmed server. Flybase (see Availability and requirements section for URL) was used to identify target genes. Candidate DT genes were distributed in different biological processes according to their gene-ontology (GO) terms [66]. This analysis was performed automatically with the aid of the program FatiGO+, from the Babelomics suite of bioinformatic tools, available at (see Availability and requirements section for URL)[67]. This program distributes the genes among the GO terms in a non-exclusive manner (i.e. a given gene might be associated to more than one GO term). Using the same program we made a comparison of the distribution of GO terms between the sample of lines that developed faster and the population of lines that developed longer in order to find GO terms that were significantly over-represented in one of the samples.

## Availability and requirements

Berkeley Drosophila Genome Project*: *

Flybase: 

FatiGO+: 

## Abbreviations

DT: developmental time; ANOVA: analysis of variance; GO: gene-ontology.

## Authors' contributions

JM and JJF conceived the study. JM, NL, VPC and AM performed the developmental time assays. JM analyzed the data and wrote the first draft of this manuscript. JM, EH and JJF wrote the final version. NL and VPC helped to draft the final version of this manuscript. All authors read and approved the final manuscript.

## Supplementary Material

Additional file 1 Correlation between viability (VT) and developmental time (DT) for all *P[GT1] *insertion lines analyzed. VT is presented in angular transformation (arcsine of the square root of the proportion of survival) and DT expressed in hours. Solid line represents the correlation plot and dashed lines the 95% confidence intervals (r = 0.305). Click here for file

Additional file 2 Mean values of significant *P[GT1] *insertion lines on DT (25°C). Values are shown as the deviation of the insertion line mean from contemporaneous control for each sex separately. Click here for file

Additional file 3 Genetic information of candidate DT genes including gene names, *P[GT1] *insertion sites, cytologial positions, biological process gene-ontologies and pleiotropy. Click here for file
