# Supplementary figures and images for: Identifying candidate genes affecting developmental time in Drosophila melanogaster: pervasive pleiotropy and gene-by-environment interaction
Source: BMC Dev Biol. 2008 Aug 8;8:78. doi: 10.1186/1471-213X-8-78 (PMC2519079; doi:10.1186/1471-213X-8-78)

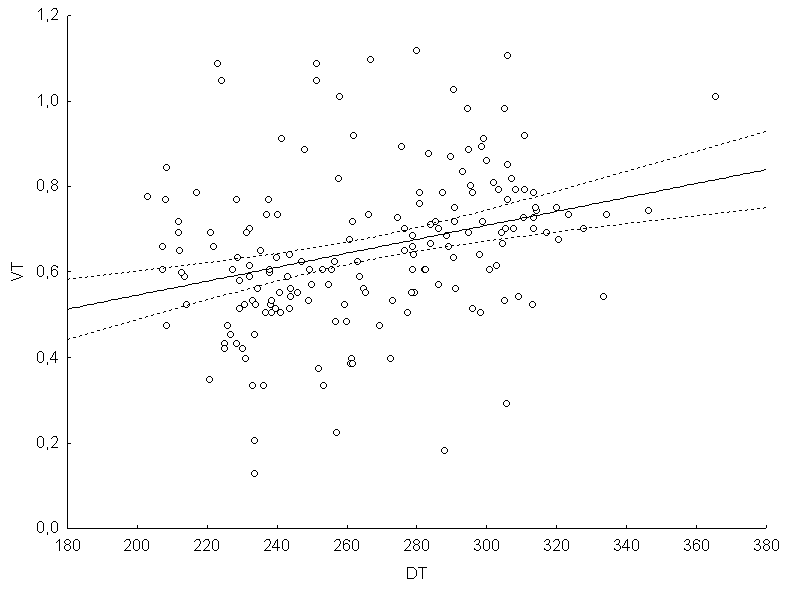

Supplement: Additional file 1 — Correlation between viability (VT) and developmental time (DT) for all P[GT1] insertion lines analyzed. VT is presented in angular transformation (arcsine of the square root of the proportion of survival) and DT expressed in hours. Solid line represents the correlation plot and dashed lines the 95% confidence intervals (r = 0.305). [file 1471-213X-8-78-S1.bmp]
