# Supplementary material for: Identifying candidate genes affecting developmental time in Drosophila melanogaster: pervasive pleiotropy and gene-by-environment interaction
Source: BMC Dev Biol. 2008 Aug 8;8:78. doi: 10.1186/1471-213X-8-78 (PMC2519079; doi:10.1186/1471-213X-8-78)
Supplement: Additional file 2 — Mean values of significant P[GT1] insertion lines on DT (25°C). Values are shown as the deviation of the insertion line mean from contemporaneous control for each sex separately. [file 1471-213X-8-78-S2.doc]

Supplementary Table 1.

Mean values of significant *P[GT1]* insertion lines on developmental time (25ºC).

| LINE | Males | Females |
| --- | --- | --- |
| BG00177 | -29.29**** | -24.68**** |
| BG00372 | 93.78**** | 108.42**** |
| BG00489 | 18.41* | 23.55** |
| BG00524 | 59.79**** | 59.09**** |
| BG00664 | 40.24**** | 56.64**** |
| BG00735 | 20.55** | 22.14**** |
| BG00846 | 79.04**** | 77.95**** |
| BG00985 | -13.54** | NS |
| BG00992 | 42.49**** | 58.88**** |
| BG01007 | 20.46** | 25.55** |
| BG01010 | 104.76**** | 116.92**** |
| BG01011 | 48.73**** | 49.61**** |
| BG01014 | 42.21**** | 41.98**** |
| BG01017 | 51.61**** | 54.43**** |
| BG01019 | 15.35** | NS |
| BG01037 | NS | 14.25** |
| BG01045 | 49.4**** | 65.47**** |
| BG01047 | -31.85**** | -29.41**** |
| BG01062 | 55.26**** | 69.12**** |
| BG01065 | -23.66*** | -27.78**** |
| BG01081 | 42.09**** | 49.54**** |
| BG01127 | 57.82**** | 81.9**** |
| BG01218 | -24.54**** | -19.86*** |
| BG01228 | -10.20* | NS |
| BG01247 | 52.64**** | 75.16**** |
| BG01257 | 15.83* | 39.5**** |
| BG01279 | 46.18**** | 58.33**** |
| BG01290 | 20.12** | 31.38**** |
| BG01297 | 23.55**** | 19.17**** |
| BG01361 | 18.01*** | 30.48*** |
| BG01412 | 119.0**** | 146.65**** |
| BG01491 | 45.36**** | 54.02**** |
| BG01498 | 16.73*** | 10.81* |
| BG01520 | 58.23**** | 48.52**** |
| BG01543 | -60.87**** | -49.77**** |
| BG01548 | 63.42**** | 77.27**** |
| BG01563 | -18.91* | NS |
| BG01565 | 26.29**** | 29.44**** |
| BG01566 | 18.79** | 19.08** |
| BG01568 | -34.18**** | -35.26**** |
| BG01573 | 22.96** | 26.63** |
| BG01600 | 60.8**** | 65.27**** |
| BG01618 | 58.66**** | 82.01**** |
| BG01649 | 66.08**** | 76.04**** |
| BG01655 | 16.00**** | 8.11* |
| BG01660 | NS | 28.84*** |
| BG01662 | -26.5*** | NS |
| BG01683 | -41.16**** | -33.68**** |
| BG01689 | 47.54**** | 57.26**** |
| LINE | Males | Females |
| BG01713 | 81.32**** | 86.51**** |
| BG01736 | 36.87**** | 45.43**** |
| BG01769 | -44.98**** | -36.28**** |
| BG01784 | 38.1**** | 40.41**** |
| BG01828 | -26.21**** | -25.92**** |
| BG01835 | 82.88**** | 97.87**** |
| BG01858 | 47.62**** | 46.72**** |
| BG01859 | 40.91**** | 56.11**** |
| BG01892 | 88.46**** | 104.18**** |
| BG01898 | -41.14**** | -39.11**** |
| BG01949 | NS | 7.71* |
| BG02023 | -21.07* | -15.14* |
| BG02034 | -14.39* | NS |
| BG02065 | 15.36** | NS |
| BG02067 | -32.07**** | -29.86**** |
| BG02095 | NS | 12.83* |
| BG02106 | 57.57**** | 68.78**** |
| BG02113 | 59.71**** | 71.32**** |
| BG02132 | NS | 9.57** |
| BG02173 | 12.44**** | 8.83* |
| BG02192 | 75.42**** | 71.14**** |
| BG02219 | -30.33**** | -28.72**** |
| BG02240 | 8.27* | 9.26** |
| BG02262 | NS | -21.37* |
| BG02286 | 57.44**** | 72.44**** |
| BG02292 | 70.82**** | 95.29**** |
| BG02327 | 27.61**** | 22.90**** |
| BG02386 | 9.54* | NS |
| BG02398 | 14.82*** | 11.40* |
| BG02439 | 21.11*** | 52.96**** |
| BG02480 | 66.79**** | 69.13**** |
| BG02546 | -18.55*** | -24.86**** |
| BG02560 | 13.94** | NS |
| BG02563 | 50.14**** | 67.38**** |
| BG02566 | 37.69**** | 46.41**** |
| BG02727 | NS | 11.27*** |
| BG02830 | 41.14**** | 78.6**** |

NS: no significant.

*p<0.05; **p<0.01; ***p<0.001: ****p<0.0001
