# Supplementary material for: Identifying candidate genes affecting developmental time in Drosophila melanogaster: pervasive pleiotropy and gene-by-environment interaction
Source: BMC Dev Biol. 2008 Aug 8;8:78. doi: 10.1186/1471-213X-8-78 (PMC2519079; doi:10.1186/1471-213X-8-78)
Supplement: Additional file 3 — Genetic information of candidate DT genes including gene names, P[GT1] insertion sites, cytologial positions, biological process gene-ontologies and pleiotropy. [file 1471-213X-8-78-S3.doc]

Supplementary Table 2.

Genetic information of candidate developmental time genes.

| |  |  |  |  |  |  | | --- | --- | --- | --- | --- | --- | | Line | Gene | *P [GT1]* insertion site | Cytological position | Biological Process  Gene ontology | Pleiotropy | |  |  |  |  |  |  | | BG00177 | *CG9894* | in gene | 22A3 |  |  | | BG00369 | *CG13333 /*  *CG13334* | 666 bp at 5´side CG13333;  1198 bp at 3´side CG13334 | 50B1-2 | / Cellular metabolic process. Macromolecule metabolic process. Catabolic process. | I | | BG00372 | *CG1678* | 1498 bp at 3' side | 20A1 |  |  | | BG00373 | *CG11226* | 1582 bp at5´side | 80D1 |  |  | | BG00386 | *Nmdar1* | 302 bp at 3´side | 83A6-A7 | Establishment of localization. | Olfactory^c^ | | BG00489 | *Osiris 9* | 153 bp at 3' side | ´83E2 |  |  | | BG00524 | *pri* | 250 bp at 3' side | 87F14 |  |  | | BG00735 | *Schizo (siz)* | in gene | 78A5-B1 |  |  | | BG00737 | *Hsp27* | in gene | 67B1 |  |  | | BG00846 | *invected* | in gene | 48A1 |  |  | | BG00992 | *CG17574* | in gene | 49D-D6 |  |  | | BG01007 | *Nemy* | 193 bp at 3' side | 16C4-D1 | Cellular metabolic process. Nitrogen compound metabolic process. Behavior. |  | | BG01010 | *Karl* | in gene | 10E3 |  |  | | BG01011 | *Misshapen (msn)* | 1402 bp at 3' side | 62E6-E7 |  |  | | BG01014 | *spichthyin (spict)* | in gene | 33F3 |  |  | | BG01017 | *CG14579* | 16156 bp at 3' side | 19F2 |  |  | | BG01028 | *Trl* | in gene | 70F4 |  |  | | BG01037 | *βv Integrin* | 127 bp at 3' side | 39A1 |  | Olfactory^c^ | | BG01045 | *Ninjurin A* | 41979 bp at 5' side | 67E7 | Multicellular organismal development. Anatomical structure development. Cell adhesion. Developmental growth. Response to external stimulus. |  | | BG01047 | *Frizzled* | 565 bp at 3' side | 70D4-5 | Multicellular organismal development. Cellular developmental process. Cell communication. Cellular component organization and biogenesis. Cell adhesion. Protein localization. | Olfactory^c^,  Starvation resistance^b^ | | BG01062 | *brother of iHog* | in gene | 3A3 |  |  | | BG01065 | *SH3PX1 /*  *Visgun* | 577 bp at 5' side: SH3PX1  201 bp at 3' side: vsg | 67C5 |  |  | | BG01081 | *Glutamate oxaloacetate transaminase 1* | in gene | 52F11-53A1 | Cellular metabolic process. Biosynthetic process. Nitrogen compound metabolic process. |  | | BG01127 | *muscleblind* | 10005 bp at 3' side | 54B1-5 |  |  | | BG01214 | *sgl* | in gene | 65D4-D5 |  | Olfactory^c^ | | BG01218 | *CG6767* | in gene | 67C4-C5 | Cellular metabolic process. |  | | BG01228 | *derailed (drl)* | 23606 bp at 5' side | 37C7 | Cellular metabolic process. Multicellular organismal development. Macromolecule metabolic process. Anatomical structure development. Cellular developmental process. Cell communication. Cellular component organization and biogenesis. Behavior. Response to chemical stimulus. Localization of cell. |  | | BG01247 | *SRY interacting protein 1* | 9283 bp at 3' side | 54B16 |  |  | | BG01257 | *1.28* | 147 bp at 3' side | 42B3 | Multicellular organismal development. |  | | BG01279 | *CG17836* | in gene | 91D4-5 |  | Olfactory^c^ | | BG01290 | *Btk family kinase at 29A (Btk29A)* | in gene | 29A1-A3 | Cellular metabolic process. Multicellular organismal development.  Macromolecule metabolic process. Anatomical structure development.  Cellular developmental process. Cell communication. Sexual reproduction. Establishment of localization. Behavior. Behavioral interaction between organisms. Reproductive process. Aging. Intercellular bridge organization and biogenesis. |  | | BG01297 | *CG33691* | 1158 bp at 3' side | 6E2 |  |  | | BG01339 | *clt* | in gene | 57F4 |  |  | | BG01354 | *CG30492* | 255 bp at 3´ side | 43E5-7 |  |  | | BG01361 | *CG6854* | in gene | 71B4-5 | Cellular metabolic process. Biosynthetic process. |  | | BG01488 | *musashi (msi)* | in gene | 96E2-4 |  |  | | BG01491 | *tramtrack (ttk)* | 1637 bp at 3' side | 100F3 |  | Starvation resistance^b^,  Bristle number^a^ | | BG01498 | *Casein kinase Iα (CKIα)* | 486 bp at 5' side | 11B11 | Cellular metabolic process. Macromolecule metabolic process. Cell communication. Response to stress. Response to endogenous stimulus. Catabolic process. |  | | BG01520 | *E2F transcription factor (E2f)* | 15455 bp at 5' side | 93E9-F1 | Cellular metabolic process. Macromolecule metabolic process. Regulation of biological process. Establishment of localization. Cell proliferation. Cell cycle. Death. Multicellular organismal development |  | | BG01543 | *Merlin* | in gene | 18E1 | Multicellular organismal development. Anatomical structure development. Regulation of biological process. Cellular developmental process. Cell communication. Cellular component organization and biogenesis. Establishment of localization. Regulation of biological quality. Developmental growth. Organ growth. Cell proliferation. | Olfactory^c^,  Starvation resistance^b^ | | BG01548 | *α-Esterase-10 (α-Est10)* | 2571 bp at 5' side | 84D9 |  |  | | BG01563 | *CG16708* | 1016 bp at 3' side | 82F11-83A1 | Multicellular organismal development. Anatomical structure development. Regulation of biological process. Cellular developmental process. Cell communication. Death. Regulation of a molecular function. | Olfactory^c^,  Starvation resistance^b^ | | BG01565 | *Defense repressor 1 (Dnr1)* | in gene | 58E9-F1 |  |  | | BG01566 | *arrest (aret)* | 6217 bp at 5' side | 33D3-5 | Cellular metabolic process. Macromolecule metabolic process. Anatomical structure development. Regulation of biological process. Cellular developmental process. Biosynthetic process. Sexual reproduction. Cell proliferation. Cell division. |  | | BG01568 | *CG32560* | in gene | 16C1-C8 | Regulation of biological process. Cell communication. |  | | BG01573 | *forkhead box, sub-group O (foxo)* | 6088 bp at 5´ side | 88A5-7 | Cellular metabolic process. Macromolecule metabolic process. Regulation of biological process. Cell communication. Cellular component organization and biogenesis. Cell proliferation. Regulation of biological quality. Aging. Multicellular organism growth. |  | | BG01600 | *CG31666* | 1207 bp at 5´ side | 22A5-8 |  |  | | BG01618 | *CG6398* | 142 bp at 3' side | 16D5-7 |  |  | | BG01649 | *cricklet (clt)* | in gene | 57F4 |  |  | | BG01655 | CG32038 | in gene | 67B7 |  |  | | BG01662 | LanA | 64 bp at 5´ side | 65A8-9 |  |  | | BG01672 | *CG14591* | in gene | 42A8 |  | Olfactory^c^ | | BG01683 | CG32572 | in gene | 15A3 |  |  | | BG01713 | 4EHP | in gene | 95E1 |  |  | | BG01716 | *paps* | 723 bp at 5´ side | 76D1 | Cellular metabolic process. |  | | BG01726 | *CG11382* | 197 bp at 3´ side | 1E4 |  |  | | BG01735 | *CG13130 / bib* | in genes | 30F5 | */* Anatomical structure development. Cellular developmental process. Sexual reproduction. Establishment of localization. Cell communication. |  | | BG01736 | CG5966 | 29 bp at 3´side | 5D1 |  |  | | BG01763 | *CG33960* | 12446 bp at 3´ side | 53C4 |  |  | | BG01769 | Beadex (Bx) | in gene | 17C3-4 |  | Bristle number^a^ | | BG01780 | *CG11226* | in gene | 80A4 |  |  | | BG01784 | *Bunched* (*bun*) | in gene | 33E5-9 |  |  | | BG01822 | *Imp* | in gene | 9F2-F4 |  | Bristle number^a^ | | BG01858 | ade5 | 2179 bp at 3´ side | 11B16 | Cellular metabolic process. Biosynthetic process. Secondary metabolic process. |  | | BG01902 | *mam* | in gene | 50C23-D3 | Multicellular organismal development. Anatomical structure development. Cellular developmental process. Cell division. |  | | BG01912 | *pxb* | in gene | 89A1-2 | Cell communication. | Bristle number^a^ | | BG01949 | *ade5/CG12717* | 127 bp at 5´side ade5 /  490 bp at 3´side CG12717 | 11B16 | Cellular metabolic process. Biosynthetic process / Cellular metabolic process. Macromolecule metabolic process. |  | | BG01990 | *CG30492* | 501 bp at 3´side | 43E5-7 |  |  | | BG02023 | *Fasciclin (Fas3)* | in gene | 36F2-4 |  |  | | BG02034 | *lilliputian (lilli)* | in gene | 23C1-3 |  |  | | BG02042 | *eas* | in gene | 14B7 |  |  | | BG02065 | *Toucan (toc)* | in gene | 23D1-2 |  |  | | BG02067 | *Aats-ile* | In gene | 79D4 | Cellular metabolic process. Macromolecule metabolic process. Biosynthetic process. Nitrogen compound metabolic process. |  | | BG02088 | *CG15309* | in gene | 9B4 |  |  | | BG02095 | *Echinoid (ed)* | 2888 bp at 3´ side | 24D4-6 |  |  | | BG02102 | *CG13434 / l(2)05510* | 1676 bp at 3´side: CG13434 /  2201 bp 5´side: l(2)05510 | 57A5-6 |  |  | | BG02106 | CG31145 | in gene | 95A4-7 |  |  | | BG02113 | *Laminin A (LanA)* | 674 bp at 5´ side | 65A8-9 | Multicellular organismal development. Regulation of biological process. Cellular developmental process. Cellular component organization and biogenesis. Cell cycle. Localization of cell . Chromosome segregation. |  | | BG02157 | *CG8177* | 2746 bp at 3´side | 67C7-8 |  |  | | BG02159 | *CG32666* | in gene | 10C1-5 | Cellular metabolic process. Macromolecule metabolic process. Anatomical structure development. Cellular component organization and biogenesis. Regulation of biological process. Cell adhesion. |  | | BG02173 | NFAT | in gene | 12A9-B2 | Cellular metabolic process. Macromolecule metabolic process. Regulation of biological process. |  | | BG02192 | *boule (bol)* | in gene | 66F5-67A1 | Cellular metabolic process. Multicellular organismal development. Macromolecule metabolic process. Regulation of biological process. Cellular developmental process. Biosynthetic process. Sexual reproduction. Cell cycle. |  | | BG02219 | *Smrter (Smr)* | 375 bp at 3´side | 11B10-14 |  |  | | BG02239 | *CG11550* | 9536 bp at 5´side | 100D1 | Establishment of localization. |  | | BG02240 | *bip1* | 4666 bp at 5´side | 66B3 |  |  | | BG02262 | *Smrter (Smr)* | in gene | 11B10-14 |  |  | | BG02286 | *Amnesiac (amn) / CG32529* | in genes | 18F4-19A2 |  |  | | BG02292 | *CG2865* | 11632 bp at 5´side | 2F4-5 |  |  | | BG02327 | *Pipsqueak (psq*) | In gene | 47A13-B1 |  | Olfactory^c^ | | BG02386 | *Semaphorin-5C (Sema-5C)* | 171 bp at 5´side | 68F2 |  | Olfactory^c^ | | BG02398 | CG41475 | in gene | 20D2 |  |  | | BG02439 | *CG32556* | 264 bp at 3´side | 16B12-C1 |  | Olfactory^c^ | | BG02462 | *CG6301* | 1398 bp at 5´side | 53D11 |  |  | | BG02480 | *CG31176* | 1406 bp at 5´side | 93F2-6 |  |  | | BG02560 | CG9674 | in gene | 76C4-D1 | Cellular metabolic process. Nitrogen compound metabolic process. Biosynthetic process. |  | | BG02563 | *capricious* (*caps*) | in gene | 70A3-4 |  | Bristle number^a^ | | BG02566 | *Calreticulin* (*Crc*) | in gene | 85E1 |  | Olfactory^c^,  Bristle number^a^ | | BG02690 | *CG14478* | in gene | 54B16 | Cellular metabolic process. Macromolecule metabolic process. |  | | BG02727 | *Escargot* (*esg*) | 110 bp at 3´side | 35D2 |  | Bristle number^a^ | | BB02747 | *rut* | in gene | 12F4 | Cellular metabolic process. Biosynthetic process. Behavior. Behavioral interaction between organisms. Reproductive process. Molting cycle. Neurological process. Response to chemical stimulus. Cell communication. | Bristle number^a^ | | BG02823 | *scyl* | 157 bp at 3´ side | 68B4-C1 | Regulation of biological process. | Olfactory^c^ | | BG02830 | *Lipid storage droplet-2* (*Lsd-2*) | 61 bp at 5´side | 13A8-9 | Multicellular organismal development. Cellular component organization and biogenesis. Establishment of localization. |  | |
| --- | --- | --- | --- | --- | --- | --- | --- | --- | --- | --- | --- | --- | --- | --- | --- | --- | --- | --- | --- | --- | --- | --- | --- | --- | --- | --- | --- | --- | --- | --- | --- | --- | --- | --- | --- | --- | --- | --- | --- | --- | --- | --- | --- | --- | --- | --- | --- | --- | --- | --- | --- | --- | --- | --- | --- | --- | --- | --- | --- | --- | --- | --- | --- | --- | --- | --- | --- | --- | --- | --- | --- | --- | --- | --- | --- | --- | --- | --- | --- | --- | --- | --- | --- | --- | --- | --- | --- | --- | --- | --- | --- | --- | --- | --- | --- | --- | --- | --- | --- | --- | --- | --- | --- | --- | --- | --- | --- | --- | --- | --- | --- | --- | --- | --- | --- | --- | --- | --- | --- | --- | --- | --- | --- | --- | --- | --- | --- | --- | --- | --- | --- | --- | --- | --- | --- | --- | --- | --- | --- | --- | --- | --- | --- | --- | --- | --- | --- | --- | --- | --- | --- | --- | --- | --- | --- | --- | --- | --- | --- | --- | --- | --- | --- | --- | --- | --- | --- | --- | --- | --- | --- | --- | --- | --- | --- | --- | --- | --- | --- | --- | --- | --- | --- | --- | --- | --- | --- | --- | --- | --- | --- | --- | --- | --- | --- | --- | --- | --- | --- | --- | --- | --- | --- | --- | --- | --- | --- | --- | --- | --- | --- | --- | --- | --- | --- | --- | --- | --- | --- | --- | --- | --- | --- | --- | --- | --- | --- | --- | --- | --- | --- | --- | --- | --- | --- | --- | --- | --- | --- | --- | --- | --- | --- | --- | --- | --- | --- | --- | --- | --- | --- | --- | --- | --- | --- | --- | --- | --- | --- | --- | --- | --- | --- | --- | --- | --- | --- | --- | --- | --- | --- | --- | --- | --- | --- | --- | --- | --- | --- | --- | --- | --- | --- | --- | --- | --- | --- | --- | --- | --- | --- | --- | --- | --- | --- | --- | --- | --- | --- | --- | --- | --- | --- | --- | --- | --- | --- | --- | --- | --- | --- | --- | --- | --- | --- | --- | --- | --- | --- | --- | --- | --- | --- | --- | --- | --- | --- | --- | --- | --- | --- | --- | --- | --- | --- | --- | --- | --- | --- | --- | --- | --- | --- | --- | --- | --- | --- | --- | --- | --- | --- | --- | --- | --- | --- | --- | --- | --- | --- | --- | --- | --- | --- | --- | --- | --- | --- | --- | --- | --- | --- | --- | --- | --- | --- | --- | --- | --- | --- | --- | --- | --- | --- | --- | --- | --- | --- | --- | --- | --- | --- | --- | --- | --- | --- | --- | --- | --- | --- | --- | --- | --- | --- | --- | --- | --- | --- | --- | --- | --- | --- | --- | --- | --- | --- | --- | --- | --- | --- | --- | --- | --- | --- | --- | --- | --- | --- | --- | --- | --- | --- | --- | --- | --- | --- | --- | --- | --- | --- | --- | --- | --- | --- | --- | --- | --- | --- | --- | --- | --- | --- | --- | --- | --- | --- | --- | --- | --- | --- | --- | --- | --- | --- | --- | --- | --- | --- | --- | --- | --- | --- | --- | --- | --- | --- | --- | --- | --- | --- | --- | --- | --- | --- | --- | --- | --- | --- | --- | --- | --- | --- | --- | --- | --- | --- | --- | --- | --- | --- | --- | --- | --- | --- | --- | --- | --- | --- | --- | --- | --- | --- | --- | --- | --- | --- | --- | --- | --- | --- | --- | --- | --- | --- | --- | --- | --- | --- | --- | --- | --- | --- | --- | --- | --- | --- | --- | --- | --- | --- | --- | --- | --- | --- | --- | --- | --- | --- | --- | --- | --- | --- | --- | --- | --- | --- | --- | --- | --- | --- | --- | --- | --- | --- | --- | --- | --- | --- | --- | --- | --- | --- | --- | --- | --- | --- | --- | --- | --- | --- | --- | --- | --- | --- | --- | --- | --- | --- | --- | --- | --- | --- | --- | --- | --- | --- | --- | --- | --- | --- | --- | --- | --- | --- | --- | --- | --- | --- | --- | --- | --- | --- | --- | --- | --- | --- | --- | --- | --- | --- | --- | --- | --- | --- | --- | --- | --- | --- | --- | --- | --- |

a, Norga *et al*., 2003. b, Harbison *et al*., 2004. c, Sambandan *et al*. 2006.
